# Effect of Wet Granulation on Tribological Behaviors of Cu-Based Friction Materials

**DOI:** 10.3390/ma16031075

**Published:** 2023-01-26

**Authors:** Lekai Li, Jian Zhuang, Tianjian Tong, Jin Tong, Xucheng Zhao, Feipeng Cao, Wei Song, Donghai Wang, Yitong Tian, Yunhai Ma, Dongyu Chen, Qifeng Zhang

**Affiliations:** 1 Key Laboratory of Bionic Engineering (Ministry of Education), College of Biological and Agricultural Engineering, Jilin University, Changchun 130022, China; 2 Weihai Institute for Bionics, Jilin University, Weihai 264200, China; 3 Department of Agricultural and BioSystem Engineering, Iowa State University, Ames, IA 50010, USA; 4 Liaoyuan Steel Back Bearing Co., Ltd., Liaoyuan 136200, China; 5 Shandong Linglong Testing Technology Co., Ltd., Yantai 264000, China

**Keywords:** granulation, tribological behavior, copper-based friction materials, wear mechanism

## Abstract

Because of the excellent thermal conduction, corrosion resistance, and tribological properties, copper-based friction materials (CBFMs) were widely used in airplanes, high-speed trains, and wind power generation. With operating speed continuously increasing, CBFMs are suffering more complicated and extreme working conditions, which would cause abnormal abrasion. This paper presents an experiment to investigate how the tribological behaviors of CBFMs are regulated by granulation technology. Samples were prepared by the method of granulation and cool-pressed sinter. The tribological properties of specimens with different granule sizes were studied. The results showed that granulation could improve the tribological properties of CBFMs. The friction coefficient (COF) increased first and then decreased with increasing granule size. Specimen fabricated with 5–8 mm granules obtained the lowest COF, which was reduced by 22.49% than that made of powders. Moreover, the wear rate decreased first and then increased as granule size increased. The wear rate of samples prepared by granules 3–5 mm was lower than that of all of the other samples. This is because the structured samples prepared by wet granulation can promote the formation of secondary plateaus, which are beneficial for enhancing tribological properties. This makes granulation a promising method for enhancing the tribological performances of CBFMs.

## 1. Introduction

Copper matrix composites (CMCs) are widely used in many fields, such as electronics, machinery, and transportation, due to their excellent electrical, thermal conductivity, and tribological properties [1,2,3]. Copper-based friction materials(CBFMs) are used in brake components such as high-speed trains, wind power generation, and aircraft and play a vital role in their safe and stable operation [4,5]. With the progress of science and technology, CBFMs will be used at a higher speed, a greater load, and other extreme working conditions, which causes a series of further highlighted problems [6]. Therefore, it is of great significance to improve the CBFMS’ tribological properties.

Many reinforcements, such as ceramic particles and graphite, have been added to CBFMs to improve the tribological properties. For example, Peng Zhang et al. [6] added granular graphite and flake graphite to CBFMs; the addition of granular graphite and flake graphite could significantly reduce the wear rate, and the ratio of 5 wt% flake graphite to 5 wt% granular graphite was fantastic for high performance. However, the addition of graphite will also reduce the friction coefficient (COF). Weiqi Lian [7] reported that Ti_3_AlC_2_ would form a continuous and dense friction layer with graphene oxide during braking, which can reduce the wear rate of CBFMs. However, it will also cause a lower COF. Haohao Zou [8] investigated the influence of SiO_2_ on the tribological behaviors of CBFMs. They reported that hard SiO_2_ particles were useful for restricting severe plastic deformation and adhesion contact during friction. From the above studies, it can be inferred that lubrication phases can reduce the wear rate. At the same time, there is a negative influence on COF. Metal and ceramic particles with high hardness, such as Cr [9], FeCr [9], and copper powder third body [10], can form the dispersion strengthened of CBFMs, thus improving their tribological properties. In addition, due to poor wetting between ceramic particles and copper matrix, copper plating on a ceramic particle surface can achieve better wetting [9].

With the development of nanomaterials, researchers began to investigate how nanomaterials regulate CBFMs’ tribological behaviors. Nano-sized silicon carbide, with excellent chemical and physical properties, was added to CBFMs, and COF was successfully increased by 30~50%; however, it had poor stability to braking speed [11]. Different from nano-sized silicon carbide, Nano-AlN could provide a more stable COF and better wear resistance which were attributed to the fact that the Nano-AlN can refine the matrix grain of CBFMs [12]. Temel Varol et al. [13] investigated the influence of nano graphite on the tribological behavior of CBFMs. The results showed that nano graphite can regulate CBFMs’ wear mechanism. The higher the content of nano graphite, the lower the wear rate. Nevertheless, the effect of nano-graphite on COF was ignored. Changshun Zhu et al. [14] reported that with the addition of 2 vol% Boron Nitride Nanosheets to CBFMs, COF reduced from 0.15 to 0.07, and wear resistance improved by over 100%. There are plenty of studies about enhancing CBFMs’ tribological properties with reinforcement materials, but little research has been conducted regarding structure design.

In this paper, CBFMs’ tribological properties were enhanced with the method of structure design, which was different from the frequently used method of reinforcement component. CBFMs with different structural parameters were prepared by wet granulation. The periodic soft/hard arrangement structure in CBFMs was obtained, and it could promote the formation of secondary plateaus, which can improve CBFMs’ tribological properties.

## 2. Materials and Methods

### 2.1. Raw Materials

The content of CBFMs in this paper is shown in Table 1.

### 2.2. Preparation of Composites

**Figure 1:**
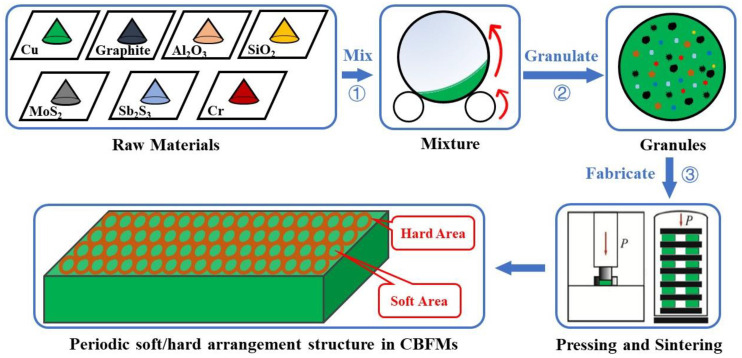
The fabrication process of CBFMs.

**Figure 2:**
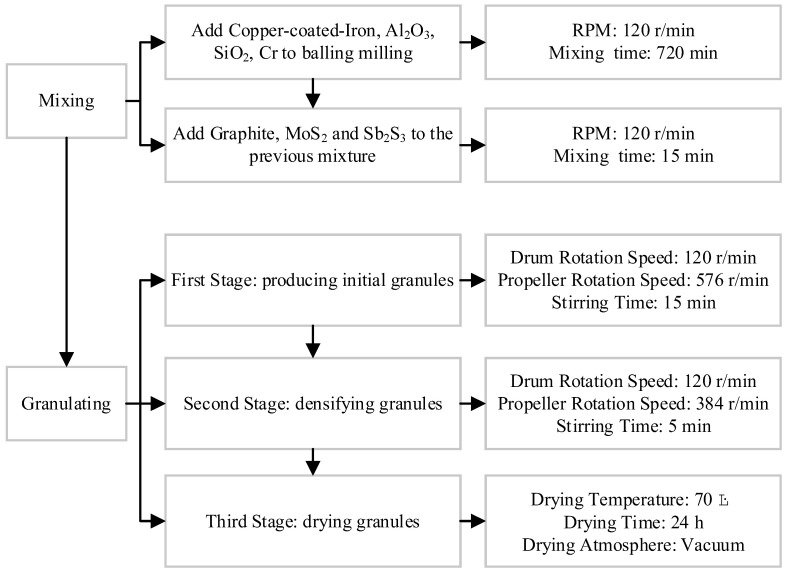
The mixing and granulating process.

The fabrication process of CBFMs is presented in Figure 1. The raw materials were mixed by the method of step feeding with ball milling (QXQM-2, Changsha Tencan Powder Technology Co., Ltd., Changsha, China), and the mixing parameters were shown in Figure 2. The ball diameters were 3, 5, and 7 mm, and the ratio of ball to powder was 10:1. During the mixing process, very few Acrylic Resin Powders (0.5 ωt%, Mitsubishi Group, Tokyo, Japan) with low molecular weight was added into the mixture of raw materials which would act the role of binder when granulating. After mixing, the mixture was granulated with laboratory tumbling granulator (JF805R, Jilin Electrical and Mechanical Equipment Co., Ltd., Changchun, China), and the technological process is shown in Figure 2. During the process of granulating, moderate absolute ethyl alcohol was sprayed into the mixture, which would bind the mixture as granules together with Acrylic Resin Powders. Moreover, there was more Acrylic Resin inside the granules and less on the surface of granules. Then, the granules were divided into four groups according to granules size. They were 1–3 mm (named GL-1), 3–5 mm (named GL-2), 5–8 mm (named GL-3), and mixed (the mixture of each granule size between 1 mm and 8 mm, named GL-4). Next, the above four groups, together with GL-0 (powders), were placed into the mold, respectively, pressed with the pressure of 600 MPa for 1 min using hydropress (LHP-5007, Liaoyuan Steel Back Bearing Co., Ltd., Liaoyuan, China). In the compacts, there would be periodic alternating arrangement areas; some of them contained more Acrylic Resin, while others contained less. Finally, the compacts were sintered in sintering furnace (69C9, Liaoyuan Steel Back Bearing Co., Ltd., Liaoyuan, China) in pure hydrogen atmosphere at 950 °C for 120 min. During sintering, the Acrylic Resin would carbonize, causing relatively soft areas and other relatively hard areas in the specimen. Thus, the periodic soft/hard arrangement structure was formed, and it was beneficial for the formation of secondary plateaus.

### 2.3. Testing Methods and Equipment

The tribological properties were tested on a friction and wear tester (JF75, Jilin Electrical and Mechanical Equipment Co., Ltd., Changchun, China). The specimens were pressed on the brake disc with a load of 4 bars, the sliding speed was 770 rpm with a friction radius of 0.108 m, and the sliding distance was 1200 m. The friction coefficient was calculated automatically by the software of machine, and the wear rate Δ*W* was determined by the following equation [15,16]:

$$∆W=\frac{1}{2\pi R}\times \frac{A}{n}\times \frac{h_{1}-h_{2}}{f}$$

where *R* (*R* = 0.108 m) is the distance between specimen and brake disc center; *A* (*A* = 176.625 mm^2^) is the area of contact surface; *n* (*n* = 1770) is the revolutions of the brake disc; *h*_1_ and *h*_2_ are the thickness of the specimen before and after tests, respectively (mm); *f* is the mean friction force during tests (N).

### 2.4. Characterization of Worn Surface

The worn surface morphologies of the specimens were evaluated using Scanning Electron Microscope (SEM, EVO-18, ZEISS, Jena, Germany) at 20 kV and Ultra-Depth Three-Dimensional Microscope (KEYENCE, VHX-6000, Xuzhou, China).

## 3. Result and Discussion

### 3.1. Friction Performance Analysis

**Figure 3:**
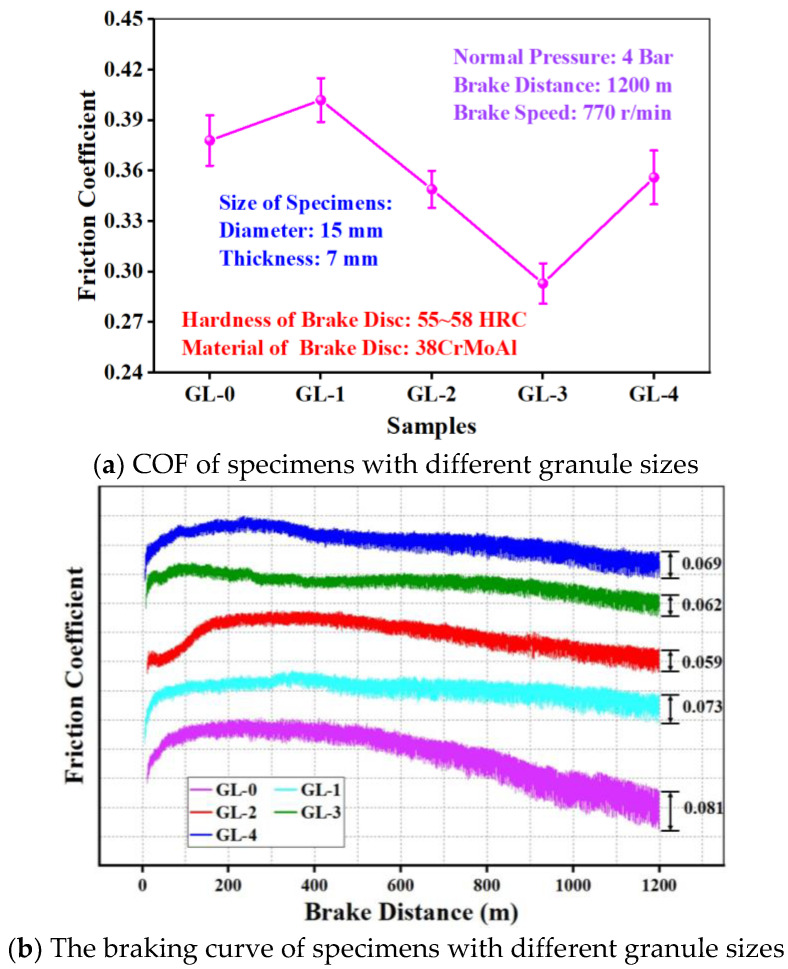
COF of specimens with different granule sizes.

Figure 3 presents the COF of specimens with different granule sizes. COF increased first and then decreased with increasing granule size, which can be effectively controlled by granulation (Figure 3a). When the granule size was small (1–3 mm), COF reached the maximum of 0.402, which was 6.35% higher than that of GL-0. With the increase in granule size, COF decreased gradually. When the granule size was 5–8 mm, COF reached the minimum of 0.293, which was 22.49% lower than that of GL-0. COF of GL-4 (mixed granule size) had a mixing tribological behavior. This is similar to our previous research [17,18].

The braking curve of all specimens is shown in Figure 3b, and they can be divided into two stages. COF increased rapidly in the first stage, while it decreased slowly in the second stage. This is consistent with the previous report [6,19,20]. In the first stage, the specimen was in direct contact with the brake disc, and COF was low. As the friction continues, the wear debris generated from the friction enters the friction surface, which impedes the relative slip between the specimen and brake disc, thus causing a rapid increase in COF [21]. With the further progress of friction, the specimen would soften so that the shear strength would decrease caused by the friction heat. In this stage, COF showed a slow decline trend [17,22].

In the second stage of braking, the COF of the specimen with different granule sizes decreased at a different rate. COF of GL-0 decreased at a much higher rate than that of others, which is mainly because the CBFMs prepared by granulation is easier to form stable and dense secondary plateaus during friction, and the secondary plateaus could stabilize the COF [21]. As the braking distance increased, the COF fluctuation of GL-0 was much higher than that of other specimens. This is because there were few strengthened nucleation sites for the formation of secondary plateaus, and the low-impact secondary plateaus would be destroyed by the shear press and normal press. Then, the wear debris would enter and leave the worn surface periodically, which would cause COF to increase and decrease largely [23]. At the same time, the specimens prepared by granulation formed periodic soft/hard arrangement structures in CBFMs. The harder areas acted as the nucleation of secondary plateaus, which hindered the movement of wear debris, and the wear debris was pressed into the softer areas under normal pressure, which formed stable and dense secondary plateaus.

In fact, although granulation does not change the components of CBFMs, it modifies the physical structure of the specimens [18]. The literature reports that wet granulation could form a hard shell on the particles’ surface, which could improve the tribological properties [24,25]. Therefore, the COF of specimens prepared by granulation had a small decreasing trend with the increase of braking distance, as shown in Figure 3b, and the fluctuation of COF was much lower than that of GL-0.

### 3.2. Wear Performance Analysis

**Figure 4:**
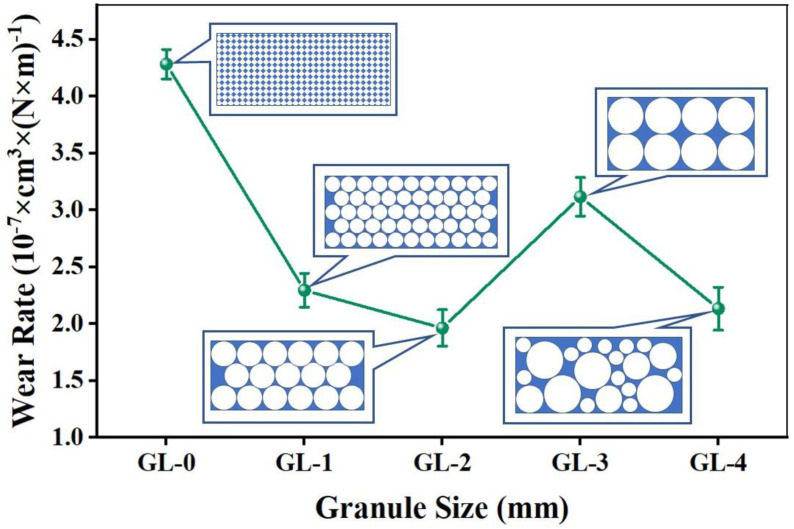
Wear rate of CBFMs.

Figure 4 shows the wear rate of each specimen. The granulation technology could efficiently regulate the wear rate of CBFMs. On the whole, the wear rate prepared by granulation has been significantly reduced by 27.2–54.2%, which is similar to our previous research [18]. For wear resistance, the sample with a granule size of 3–5 mm had the lowest wear rate, followed by specimens with a 1–3 mm granule size, and the highest wear rate was the sample with a 5–8 mm granule size.

The improvement of wear resistance depends on the protection of secondary plateaus [23,26,27]. During friction, the secondary plateaus can play a role in isolating CBFMs from the brake disc, thus reducing the abrasion [23]. The specimens prepared by granulation will periodically form a soft/hard arrangement structure in CBFMs. The hard structure becomes the nucleation site of the secondary plateaus [28], and the soft structure around the hard structure helps wear debris to be pressed in easily and promotes the formation of secondary plateaus. As shown in Figure 4, when the particle diameter is 3–5 mm, the wear rate of GL-2 was reduced by 54.2% than that of GL-0, which was mainly due to the dense secondary plateaus formed on the friction surface during braking, which prevented the wear of CBFMs.

Overall, it can be seen from the results in Figure 3 and Figure 4 that a medium granule size provided the best compromise between COF and wear rate. The tribological properties of CBFMs prepared by granulation were significantly influenced by granule size. Too small granules will cause uneven composition distribution due to their size, which deteriorates the macroscopic tribological properties of the specimens. On the other hand, too large granules, although they can ensure the macroscopic uniformity of composition, will greatly reduce the hard-to-soft area ratio in the specimen, which is disadvantageous for the formation of secondary plateaus and causes the reduced tribological properties.

### 3.3. Morphology of Worn Surface

**Figure 5:**
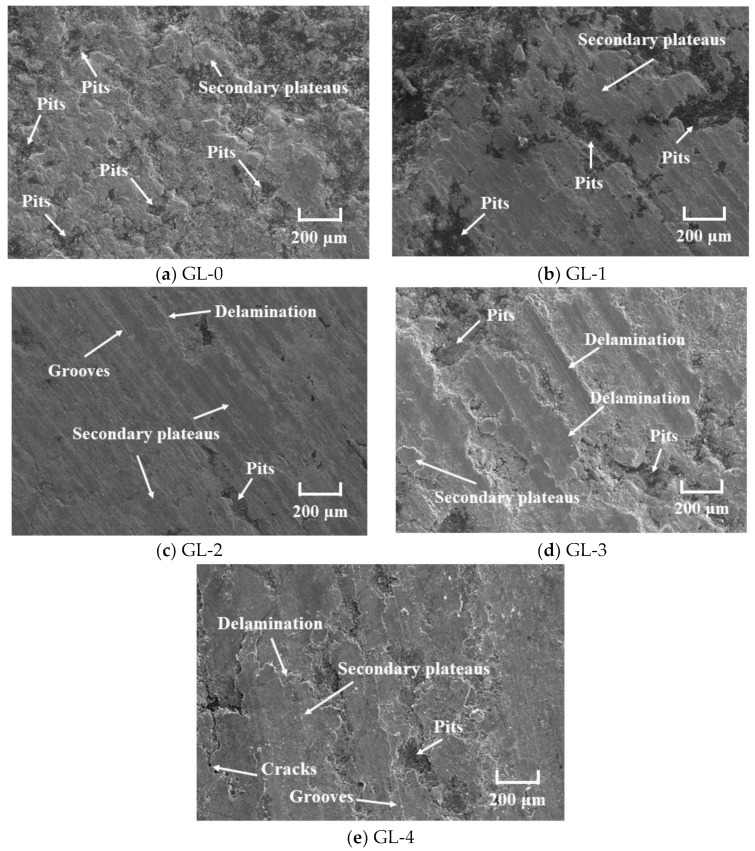
SEM micrographs of worn surfaces.

The tribological properties of friction materials are closely related to their worn surface characteristics [29,30]. It is reported that the study of the surface morphology of friction materials has become an effective tool for analyzing and explaining tribological behavior [31]. The worn surfaces SEM micrographs of five specimens are shown in Figure 5.

Figure 5a shows the worn surface morphology of GL-0. As presented in SEM micrographs, GL-0 showed the roughest worn surface. Much of the pits appeared on the worn surface, and there were also little secondary plateaus. The wear mechanism of GL-0 was severe adhesive wear, corresponding to the high wear rate of GL-0. Because of the lack of nucleation points with sufficient strength [18], it was difficult for a sample of GL-0 to form dense secondary plateaus in the friction process [21]. With the progress of friction, the copper matrix softened, and the strength decreased by the continuously increasing temperature of the worn surface. When the shear stress was higher than the binding strength, severe adhesive wear occurred [13].

**Figure 6:**
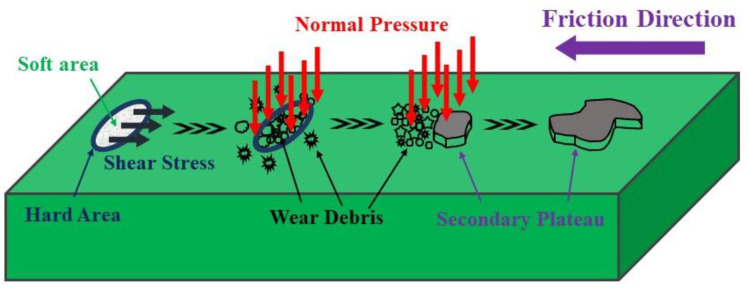
The schematic diagram of secondary plateaus for formation and growth.

The worn surface morphology of the specimen prepared by granulation is shown in Figure 5b–f. Their worn surface was smoother than that of GL-0. The worn surface morphology varied according to granule size. When braking, the specimens prepared by granulation will periodically form a soft/hard arrangement structure [18]. Because of the low strength of soft areas, the surface materials will be detached in the initial stage and cause the generation of wear debris, which will cause micro-geometric unevenness on the worn surface. At the same time, the hard areas will become the nucleation site of wear debris that accumulates on the micro-geometric unevenness. Moreover, the wear debris is compacted by normal pressure, forming dense secondary plateaus [23,28]. The secondary plateaus will lead to a sharp decrease in the actual contact area, resulting in a rapid increase in local pressure and temperature, which will densify them and strengthen their binding with the matrix [21]. The wear debris will continuously accumulate around these platforms to form new secondary plateaus, which make them continue to grow. Moreover, the actual load on these secondary plateaus will decline so that the tribological behavior gradually becomes stable [21,23]. The schematic diagram of the formation and growth of the secondary plateaus is shown in Figure 6.

As shown in Figure 5b, plenty of large secondary plateaus and discontinuous peeling pits appeared on the worn surface, which was ascribed to the imbalance of friction of different parts of the sample [17]. This is because when the granule size is small, the composition distribution is not uniform. With the increase of granule size (3–5 mm), as shown in Figure 5c, there were large continuous secondary plateaus on the worn surface that were the smoothest. Moreover, there were also shallow grooves and slight plastic deformation on the worn surface, which indicated that the wear mechanism of GL-2 was slight adhesive wear and abrasive wear [13]. This has similar results to our previous study [17]. On the one hand, the composition distributed uniformly in CBFMs as granule size was 3–5 mm, which makes the friction balance in different parts; on the other hand, the optimal proportion of soft/hard area can promote the formation of continuous and dense secondary plateaus which makes the friction more stable (corresponding the reduction of friction instability in Figure 3b) and reduce the wear rate. However, as the further increase of granule size, the proportion of soft/hard area continues to increase, and there are not enough nucleation sites on the worn surface for forming secondary plateaus, resulting in serious plastic deformation, as shown in Figure 5d. Moreover, the worn surface of the specimen prepared with mixed granules has mixed characteristics, shown in Figure 5e.

It is reported in the literature that the morphology of the counterpart surface can reflect useful information about the tribological properties of CBFMs [7,32]. According to Weiqi Lian [7], different wear mechanisms of CBFMs correspond to different morphology characteristics of counterpart surfaces. The surface of the counterpart would be covered with some large patches if CBFMs underwent adhesive wear and typical two-body abrasion wear; if severe fatigue wear occurred, there were no obvious patches on the surface of the counterpart. In our future study, we will characterize the microscopic morphology of the counterpart surface to master the relationship between the granule size of CBFMs and the morphology of the counterpart, which can integrally investigate the tribological properties of CBFMs.

### 3.4. Analysis of Worn Surface Roughness

**Figure 7:**
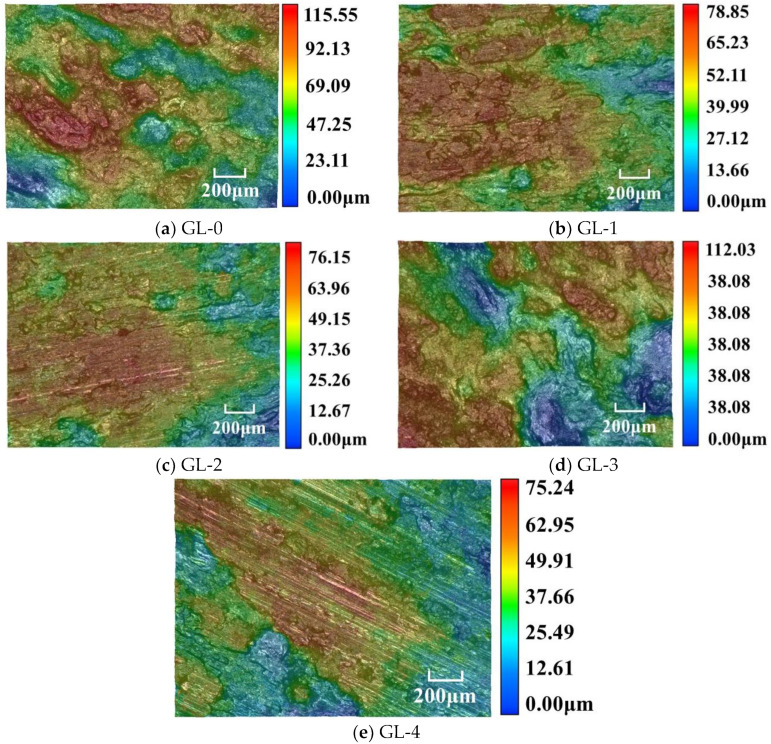
Three-Dimensional Microscope of worn surface.

The 3D contour of the worn surface for each specimen was analyzed by an Ultra-Depth Three-Dimensional Microscope, as shown in Figure 7. The worn surface of GL-0 is the roughest (Figure 7a). There are many discontinuous humps and dents on the worn surface, which is consistent with the tribological analysis results (Figure 5a). With the increase in granule size, the worn surface becomes flatter and flatter, which is similar to the results obtained by Yucheng Liu [18]. (Figure 7b,c,e). The flat areas were secondary plateaus, which correspond with the result in Figure 5b,c,e. As the granule size continued increasing (5–8 mm), the worn surface became rougher (shown in Figure 7d). Generally speaking, the higher roughness is mainly caused by the serious abrasion in the friction test [33]. The worn surface of CBFMs fabricated by granulation is smoother than that of powders, and the granulation process can enhance the wear resistance of CBFMs.

## 4. Conclusions

In this study, CBFMs were fabricated by wet granulation, and the relationship between tribological behavior and granule size was systematically investigated. Based on the above result, the main conclusions can be summarized as follows:

1. Granulation technology can effectively control the COF of CBFMs. With the increase in granule size, the COF increased first and then decreased.
2. Granulation technology can improve the COF stability of CBFMs, including heat fade resistance and friction stability.
3. Granulation technology can significantly enhance the wear resistance of CBFMs. The wear rate of GL-2 was 54.2% lower than that of GL-0.

In this paper, instead of the previous components design, tribological properties of CBFMs are improved by structure design. The method proposed in this paper provides a new idea for enhancing tribological behaviors and lays a theoretical foundation for the structural design of composite materials.

## Figures and Tables

**Table 1:** The experimental material.

Designation	Content (by wt %)	Particles Size (Mesh)	Purity	Manufacturer
Copper-coated Iron Powder	71.5	300	99%	Henan Taihe Huijin Powder Technology Co., Ltd., (Jiaozuo, China)
Graphite	11	100	99%	Shanghai Youmo Composite Materials Co., Ltd., (Shanghai, China)
MoS_2_	5	200	99.8%	Nangong Chunxu Metal Material Factory Co., Ltd., (Xingtai, China)
Sb_2_S_3_	5	200	99%	Zhongke Yanuo (Beijing) Technology Co., Ltd., (Beijing, China)
Al_2_O_3_	3	100	99.9%	Suiye Electronic Applied Materials Co., Ltd., (Shanghai, China)
SiO_2_	0.8	1000	99.4%	Hebei Keze Metal Materials Co., Ltd., (Xingtai, China)
Cr	3.7	2000	99.95%	Nangong Xindun Alloy Electrode Spray Co., Ltd., (Xingtai, China)

## Data Availability

The datasets generated and/or analyzed during the current study are available from the corresponding author upon reasonable request.
